# Space use by giant anteaters (*Myrmecophaga tridactyla*) in a protected area within human‐modified landscape

**DOI:** 10.1002/ece3.5911

**Published:** 2020-07-13

**Authors:** Alessandra Bertassoni, Guilherme Mourão, Rita de Cassia Bianchi

**Affiliations:** ^1^ Programa de Pós‐Graduação em Ecologia e Evolução Instituto de Ciências Biológicas Universidade Federal de Goiás (UFG) Goiania Brazil; ^2^ Instituto de Pesquisa e Conservação de Tamanduás no Brasil – Projeto Tamanduá Parnaíba Brazil; ^3^ Embrapa Pantanal Corumbá Brazil; ^4^ Departamento de Biologia Aplicada à Agropecuária Faculdade de Ciências Agrárias e Veterinárias Universidade Estadual Paulista (UNESP) São Paulo Brazil; ^5^ Programa de Pós‐Graduação em Biologia Animal Instituto de Biociências Letras e Ciências Exatas Universidade Estadual Paulista (UNESP) Sao Jose do Rio Preto Brazil

**Keywords:** anthropogenic site, Global Positional System, Pilosa, savanna, spatial ecology, xenarthra

## Abstract

1. Spatial ecology data are essential for conservation purposes, especially when extinction risk is influenced by anthropogenic actions. Space use can reveal how individuals use the habitat, how they organize in space, and which components are key resources for the species.
2. We evaluated the space use and multiscale habitat selection of giant anteaters (*Myrmecophaga tridactyla*), a vulnerable Neotropical mammal, in a Cerrado site within a human‐modified landscape in southeastern Brazil.
3. We used GPS transmitters to track eight anteaters in the wild. With the resulting dataset, we estimated home range and core‐area sizes and then used two overlap indexes. We assessed habitat selection by compositional analysis and analyzed events of spatio‐temporal proximity.
4. The average Brownian bridge kernel estimate of home range size was 3.41 km^2^ (0.92–7.9). Regarding home range establishment, five individuals showed resident behavior. Males (*n* = 4) had larger home ranges and were more active than females (*n* = 4). Despite the spatial overlap of home range (above 40% in four dyads), maximum temporal space sharing was 18%. Giant anteaters were found in proximity. Habitat selection favored savanna, and exotic timber plantation was always avoided. Roads and built‐up areas were selected secondarily at the landscape level.
5. The selection of anthropogenic sites denotes behavioral plasticity regarding modified habitats. However, the high selectivity for savanna, at all levels, demonstrates a high dependence on natural habitats, which provide the necessary resources for the species. The recurrent proximity of male–to‐female anteaters may indicate reproductive behavior, which is essential for maintaining this isolated population.

## Graphical abstract

We assessed space use and habitat selection of giant anteaters in a Brazilian human‐modified landscape, found the species selected for savanna and human‐related habitats.

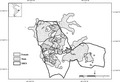

## 1 INTRODUCTION

Biotelemetry devices allow researchers to develop and test hypotheses about wild animals on a global scale. Applied to animal space use (or animal movement), these technologies have improved our insights on conservation issues (Kays, Crofoot, Jetz, & Wikelski, 2015; Martin, Tolon, Moorter, Basille, & Calenge, 2009; McGowan et al., 2017; Silva, Crane, Suwanwaree, Strine, & Goode, 2018; Tucker et al., 2018). Global Positioning System (GPS) has been used in wildlife studies since the 1990s (Tomkiewicz, Fuller, Kie, & Bates, 2010) and is now a proven tool for better understanding animal space use. This advancement has substantially improved research on home range, habitat use and selection, movement and activity patterns, and social interaction by tagged individuals (Cagnacci, Boitani, Powell, & Boyce, 2010; Kays et al., 2015).

Home range is a long‐standing concept that describes an animal's restriction of its movements to finite areas over a measurable time frame (Kie et al., 2010). More recently, home range has been conceptualized as a perceptual map, a representation of how the animal invests in and takes advantage of space (Powell & Mitchell, 2012). This space is delimited by daily movements (Calenge, Draya, & Royer‐Carenzia, 2009; Nathan, 2008) within a landscape of biotic and abiotic features, characterizing the habitat. The habitat defines the available range of resources and living conditions for a species (Hall, Krausman, & Morrison, 1997). Habitat selection is a process by which animals choose specific habitat components within the space; this process is highly scale‐dependent (i.e., position, home range scale, landscape scale; Martin et al., 2009). Therefore, understanding the spatial relationships between animals and their habitat is a central question in ecology and a critical issue for conservation management, particularly for species threatened with extinction (Falconi, Vieira, Baumgarten, Faria, & Giné, 2015; Tucker et al., 2018).

Currently, extinction rates are exceptionally high, mainly due to human population growth and increased per capita consumption (Ceballos et al., 2015; Munguía, Trejo, González‐Salazar, & Pérez‐Maqueo, 2016), both of which accelerate land‐use changes that affect ecosystems. While biodiversity conservation depends deeply on protected areas and unprotected natural remnants (Heywood & Hunter, 2010), most species that persist must do so within modified habitats (Munguía et al., 2016). It is therefore critical to understand space use in human‐modified habitats (Kays et al., 2015; McGowan et al., 2017; Munguía et al., 2016).

**Figure 1:**
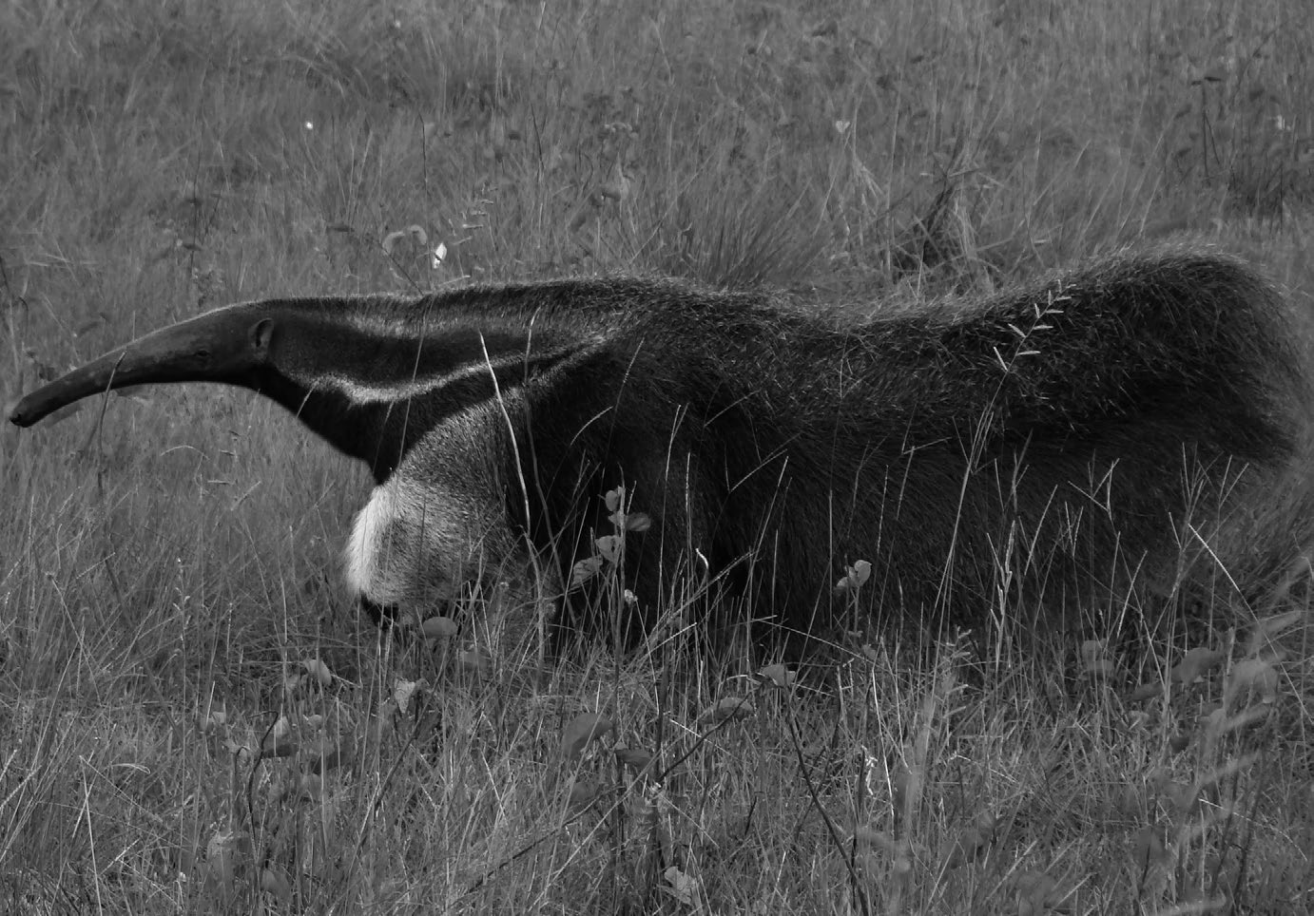
Giant anteater (*Myrmecophaga tridactyla*) by Alessandra Bertassoni

The giant anteater (*Myrmecophaga tridactyla* Linnaeus 1758; Figure 1) is considered “Vulnerable” according to the IUCN Red List (Miranda, Bertassoni, & Abba, 2014) and the Brazilian list of endangered mammals (Medri & Mourão, 2008). Over the past two decades, populations of the species have experienced regional declines (Cherem, Simões‐Lopes, & Graipel, 2004; Fallabrino & Castiñeira, 2006; Fontana, Bencke, & Reis, 2003; IAP, 2010; Passamani & Mendes, 2007), and it is considered to be extinct in Belize, Costa Rica, and Guatemala (Miranda et al., 2014). The main factors influencing its decline are habitat loss resulting from land‐use change, wildfires, overhunting, dog conflicts, and road‐kills (Miranda et al., 2014, 2015).

Habitat fragmentation can pose a major threat to anteater populations (Zimbres et al., 2013) because these insectivorous mammals require large areas for survival (up to 32.5 km^2^, Di Blanco et al., 2017). Most of our current knowledge about the use of space by giant anteaters is limited to research conducted in protected areas in Brazil and Venezuela (Camilo‐Alves & Mourão, 2006; Desbiez & Medri, 2010; Macedo, Azevedo, & Pinto, 2010; Miranda, Tomas, Valladares‐Padua, & Rodrigues, 2006; Montgomery & Lubin, 1977; Mourão & Medri, 2007; Shaw, Machado‐Neto, & Carter, 1987; Vynne et al., 2011).

Extinction proneness is related to a species' ability (or lack thereof) to adjust their behavior and use anthropogenic landscapes (Falconi et al., 2015; Terborgh, 1974); therefore, we must evaluate space use patterns not only in protected areas, but also within human‐modified landscapes. When we can compare baseline data to what we see in modified habitats, we can evaluate conservation strategies for a particular population and better understand adaptations of species overall to the Anthropocene. Such information could be essential for achieving the Aichi Biodiversity Target number 12 (to prevent the extinction known threatened species; CBD, 2011). In this paper, we study space use and habitat selection, at different scales, of giant anteaters in a 67 km^2^ study site consisting of a protected area (27 km^2^) and its human‐modified surroundings in southeast Brazil. We hypothesized that the giant anteaters living at the study site would be highly dependent on the natural features of the protected Cerrado remnant because of their extreme specializations and habitat requirements, and we therefore predict that home ranges will be primarily located within the protected area and the resource inside it will be more used than the surroundings. In addition, we also predicted that the home ranges will overlap more than 50% due to the protected area size. Our aims were to (a) estimate home range size of giant anteaters; (b) evaluate movement patterns, home range overlap and proximity of individuals, and (c) analyze and compare individuals' activity and habitat selection at scale of home range area within the landscape, and at the scale within the home range.

## 2 MATERIALS AND METHODS

### 2.1 Study site

**Figure 2:**
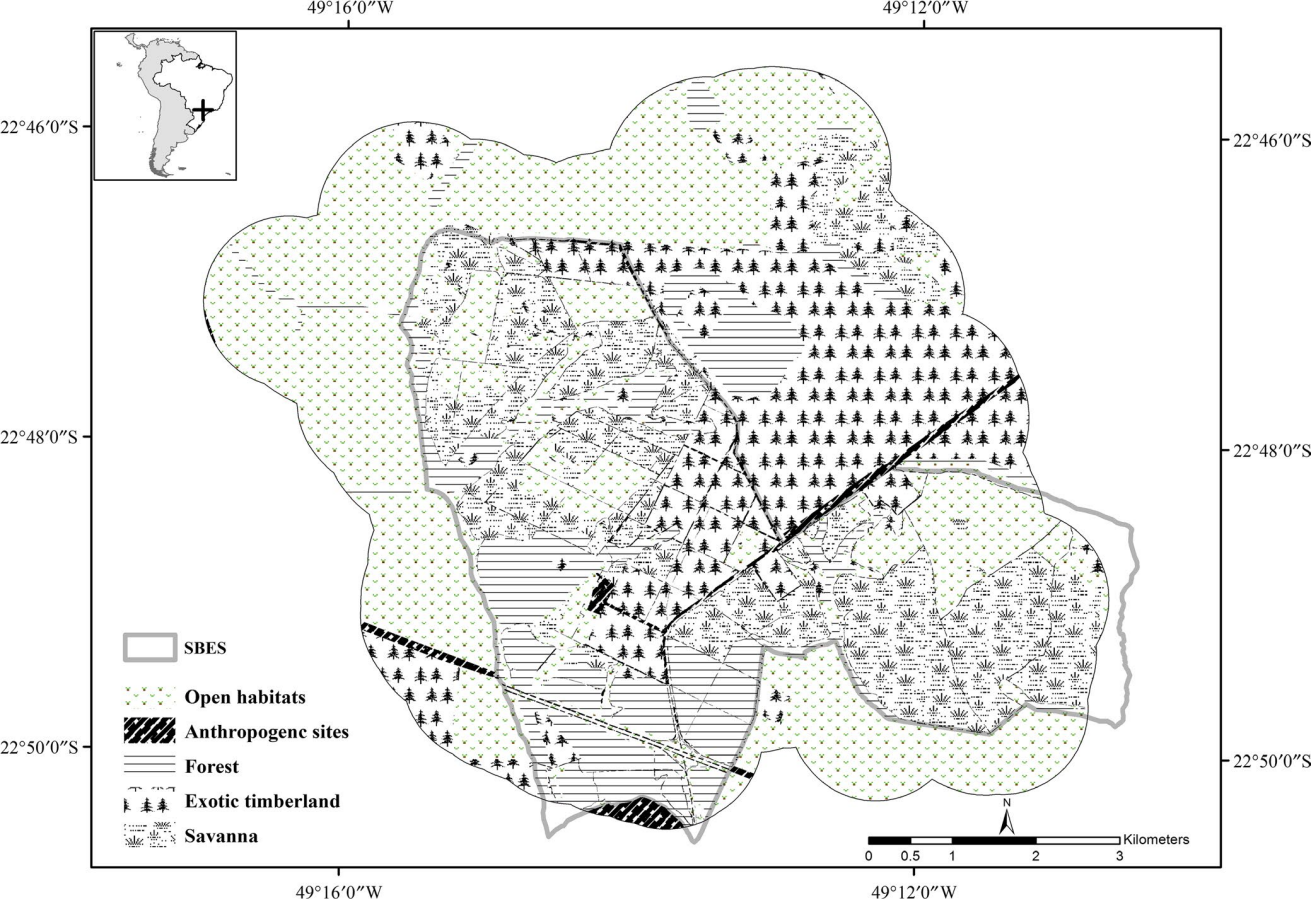
Santa Bárbara Ecological Station and its surroundings, southeast Brazil

This study took place in the Santa Bárbara Ecological Station (SBES, 22°48′59″, 49°14′12″), a protected Cerrado remnant (27 km^2^) surrounded by human‐modified landscape (Figure 2) in São Paulo State, southeastern Brazil. The Brazilian Cerrado, a tropical savanna biodiversity hotspot (Mittermeier, Myers, Mittermeier, & Robles, 1999), once covered 14% of São Paulo State; however, it has been fragmented into thousands of remnant patches through the conversion of the biome into pasture, soybean, sugarcane plantations and other perennial crops, exotic timber plantations, and urban zones (Durigan, Siqueira, & Franco, 2007). One of these patches is the SBES, which has typical Cerrado vegetation (open savanna—*campo cerrado*, shrub savanna—*cerrado denso*, forest–savanna—*cerradão*), and also has semideciduous and riparian forest and plots of exotic *Pinus* and *Eucalyptus* species. SBES is divided into four blocks by a road network comprising the SP‐280 highway, an unpaved road named SP‐261, and a dirt road that provides access to surrounding properties (Melo & Durigan, 2011). Due to the presence of open savanna physiognomies, the SBES is considered to have high biological relevance (Melo & Durigan, 2011).

The vicinity of the SBES comprises a mosaic of multiple land uses dominated by cattle ranches, agriculture, and urban areas, and it neighbors the Santa Bárbara State Forest (17 km^2^), a *Pinus* and *Eucalyptus* timber plantation owned by the São Paulo government.

The climate is *Cwa* Köppen, with monthly mean temperatures varying between 16°C and 24°C, and the elevation ranges from 600 to 680 m (Melo & Durigan, 2011).

### 2.2 Capture and tracking schedule

We carried out three capture campaigns, each one lasting about 30 days (October 2014, January and May 2015). We actively searched for individuals inside and outside the protected area, covering our entire study site of about 67 km^2^. Our surveys were conducted from a vehicle traveling 15–20 km/hr; in total, we drove about 12,600 km searching for the anteaters. Every time we detected a giant anteater, we manage to catch it using a dart‐gun, blowpipe, or Ketch All pole and net, depending on the situation. After physical restraint, we sedated each anteater with a combination of 10 mg/kg of ketamine hydrochloride, 0.8 mg/kg of xylazine hydrochloride, and 0.2 mg/kg of midazolam, giving us time to take biometric measures and attach the tracking device. All the captures were carried out in the presence of a veterinarian and followed the Guidelines for Capture, Handling and Care of Mammals of the American Society Mammalogists (Sikes & Gannon, 2011).

Followit Tellus^®^ Small (Followit AB) GPS and VHF transmitters were attached to leather harnesses which were manufactured following the guidance of Rodrigues et al. (2003) and Di Blanco, Pérez, Díaz, and Spørring (2012). The tracking package had a mass of 876 ± 3 g, corresponding to less than the 5% of body mass, as recommended (Sikes et al., 2011). We programmed the GPS to take 21 fixes per day (one each 1 hr09). Twice a month, one of us (A.B.) tracked the individuals to visually check their harnesses and their health. Until the end of the study, all the fitted animals were recaptured and had their harnesses removed.

This study was approved by the ethics committee of the School of Agricultural and Veterinarian Studies of São Paulo State University (nº 003414/13) and was performed under License SISBIO 38326‐5 (Chico Mendes Institute for Biodiversity Conservation).

### 2.3 Home range size

We calculated the size of the anteaters' home ranges by three methods: Brownian bridge kernel (BBK; Horne, Garton, Krone, & Lewis, 2007), minimum convex polygon (MCP; Burt, 1943), and fixed kernel (FK; Worton, 1989). BBK is a probabilistic estimate of an animal's trajectory that account for temporal autocorrelation between locations, as those taken by GPS (Fischer, Walter, & Avery, 2013; Silva et al., 2018). MCP and FK were developed for use with VHF data, not autocorrelated GPS data, and we are also providing them due to its use on earlier studies. Also, MCP can provide visual confirmation of home range stabilization that the other two methods cannot. In the BBK, the tracking data's temporal structure and movement patterns are incorporated, making this estimator more appropriate and biologically meaningful (Benhamou & Riotte‐Lambert, 2012; Buchin et al., 2015) than former analyses (MCP and FK). All analyses were performed in R (R Core Team, 2014).

For BBK and FK, we applied 95% and 50% of the density probability to delimit the home range and core‐area, respectively. We used the function “kernelbb” to estimate the utilization of distribution by BBK method (Calenge, 2006). The smoothing parameter that controls the kernel bandwidth along the path between successive locations (related to the animal speed), known by sig1, was estimated by maximum likelihood using the function “liker.” The smoothing parameter sig 2, which control kernel bandwidth on the relocations (related to the imprecision of the relocations), was defined as the mean value of the GPS transmitters' error. For access this value, we estimated the GPS device' error in the field, setting 10 devices in a stationary position for 12 hr to record a location every 1 hr09. Then, we calculated the mean Euclidean distance to the location and used sig2 as the standard deviation (*SD* = 15 m). The grid size was 250, which was estimated by iterations (Calenge, 2006).

To estimate the distribution of utilization by FK method, we applied the function “kernelUD” and the reference method to estimate the smoothing parameter “h.” In addition, we supplied estimates of 100% MCP using the function “mcp.area.” Data associated with the first and last days of tracking were excluded to avoid any capture bias regarding the movement of the anteaters.

The stabilization of home range area was computed based on daily accumulation of locations to the cumulative MCP home range size over the monitoring period. The estimators based on density estimation and trajectory, such as FK and BBK, are not suitable to calculate home range stabilization because their probabilistic nature causes the estimated area to decrease with the increment in locations.

### 2.4 Overlap and proximity

We used two indices to estimate home range overlap. The first, probability of home range overlap (PHR), gives the probability of animal *j* being located in animal *i's* home range (Fieberg & Kochanny, 2005). The second index, utilization distribution overlap index (UDOI), represents the joint distribution of the two animals' space use and assumes that the space is used independently. A UDOI of zero means that the two home ranges do not overlap, and if the UDOI equals one and both UDs are uniformly distributed, it means a 100% overlap. When the two UDs are not uniformly distributed, the UDOI values can be larger than one (Fieberg & Kochanny, 2005). As these analyses were based on static overlap, we used the 95% kernel estimator because it is not trajectory‐based. In addition, we examined whether the UDOI overlap varied with the sex composition of the dyads. However, because we had just three dyads composed just by females, we merged this set with the set of dyads composed just for males and applied a Kruskal–Wallis test to determine whether the UDOI overlaps of same‐sex dyads differed from the overlaps of opposite‐sex dyads.

The proximity analysis was performed to verify whether two giant anteaters were located in an area simultaneously in time and space. We compared the temporal sequence of the trajectories, using the function “GetSimultaneous” from the R package wildlifeDI (Long, Nelson, Webb, & Gee, 2014) and “Prox” from the package adehabitatHR (Calenge, 2006), to verify spatial synchrony in neighboring individuals. The average distance between consecutive locations was about 126 m. Therefore, we consider it reasonable to assume that if the distances between two anteaters were less than 300 m, this could be considered close, in the sense that if they move in the right direction, they will encounter each other within the range of 1 hr09.

### 2.5 Landscape characterization and habitat selection

We classified the vegetation types (herein considered habitat types) available in the study area into five categories according to tree coverage and the vegetation composition using the predefined categories available on the SBES planning document (Table 1) and a georeferenced digital database provided by the Forest Institute of São Paulo (Melo & Durigan, 2011). The categories were forest, exotic timber plantation, savanna, open habitats, and anthropogenic sites (Table 1). Forest includes those areas without a grass stratum and with a canopy cover of at least 80%. Exotic timber plantation refers to plots of *Pinus* spp. and *Eucalyptus* spp.. Savanna is a mix of shrub habitats with discontinuous grass stratum and tree coverage of 20%–70%. Open habitats are mainly grass‐dominated habitats, and anthropogenic sites are built‐up areas and roads. For details of vegetation physiognomies, see Durigan and Ratter (2006).

**Table 1:** Habitat categories and percentages (%) from Santa Bárbara Ecological Station and its surroundings, southeast Brazil

Habitat category	Vegetation physiognomies	%
Forest	*Cerradão*, seasonal and alluvial forests, and its transition areas	13
Exotic timber plantation	Plots of *Pinus* spp. and *Eucalyptus* spp.	22
Savanna	*Cerrado stricto *sensu and dense *cerrado*	21
Open habitats	*Cerrado* grassland and humid grassland	41
Anthropogenic sites	Roads, built‐up areas, and urban	3

Our habitat selection analysis corresponded to the 2nd and 3rd orders proposed by Johnson (1980); the former is the home range area selected by each anteater in the study area (landscape‐scale selection), and the latter represents their use within home range. The study area was delimited by a buffer made around the polygon containing the BBK‐estimated home ranges of all anteaters. The buffer (1.04 km) corresponds to the radius of the average home range size (3.41 km^2^) estimated by the BBK method. This procedure reduced the subjectivity in defining the study area (Manly, McDonald, Thomas, McDonald, & Erickson, 2004). Thus, our study site had a total area of 67 km^2^. We performed this analysis using both 95% and 50% home range estimates.

To calculate the proportion of each category within the study area, we used the R package “raster” (Hijmans, 2015) and ArcGis 10.4 (ESRI, 2016). We analyzed habitat selection using a compositional analysis (Aebischer, Robertson, & Kenward, 1993) in the R package “adehabitatHS” (Calenge, 2006), where we applied the Wilks (*λ*) test with 5,000 permutations. When the test is significant, a classification matrix of habitat selection is provided. Symbols indicate that the habitat corresponding to the line was more (+) or less (−) used than the habitat corresponding to the column. Triple symbols indicate that the habitat was not used randomly (*p* < .05). The last column shows the order of habitat usage. We conducted *eigen*‐analysis of the selection ratio, which assigns scores for each animal and habitat (Calenge & Dufour, 2006), thus enabling a graphic representation of habitat selection.

The compositional analysis was performed using all the locations in the dataset, for both the 2nd and 3rd orders of selection (sensu Johnson). Additionally, we applied the method using only the locations where the individuals were active, indicated by the GPS activity sensor. Considering the magnitude of the GPS locations error, we considered an animal inactive when consecutive locations were up to 60 m apart. This threshold was chosen based on the GPS error (15 m) and the possibility that the error could be in any of the four main directions (north, south, east, and west).

## 3 RESULTS

In total, nine giant anteaters were captured over the three monthly campaigns. The first capture occurred in January 2015 and the other eight in May–June 2015. We captured four females (F1–F4) and five males (male, M1–M4; Table 2; Appendix S1). Therefore, the female: male ratio of our dataset was 1:1.25, equivalent to 55% males.

**Table 2:** Capture dates (Start) and equipment removal (End), weight (*W*; kg), head–tail length (length; cm), and destination of giant anteaters (*Myrmecophaga tridactyla*) in 2015 in the Santa Bárbara Ecological Station and its surroundings, southeast Brazil

ID	Start	End	*W*	Length	Destination
F1	27 Jan	05 Feb	32.0	124	Unknown
M1	27 May	20 Oct	38.7	127	SBES
F2	31 May	15 Jul	34.8	129	Death
M2	02 Jun	15 Oct	35.2	132	SBES
M3	04 Jun	02 Sep	36.6	127	SBES
Male^a^	06 Jun	07 Jun	38.0	130	Death
F3	07 Jun	22 Sep	33.0	136	SBES
M4	09 Jun	23 Sep	36.2	127	SBES
F4	13 Jun	01 Sep	21.6	114	SBES

Abbreviations: F, female; M, male.

a Data of male were not included in the analysis due to his destination.

The GPS device of the female F1 failed 10 days after the capture, and its data were excluded from overlap and proximity analyses. More details about this female are given in Bertassoni et al. (2017).

### 3.1 Locations, home range, and activity

Altogether, 13,170 GPS locations were recorded on an average of 91 tracking days (Table 3; Appendix S2). Three females (F2, F3, and F4) and two males (M1 and M3) were almost exclusively SBES residents (>90% of locations). For the males M2 and M4, respectively, 21% and 42% of their locations were outside SBES; and for the female F1, 25% of her locations were outside SBES.

**Table 3:** Tracking period in days (TP), number of locations (Loc), percentage of active (Act) and inactive (Inact) locations, average distance between consecutive locations in meters (Dist), home range sizes measured (km^2^) by minimum convex polygon (MCP), fixed kernel 50% (FK50) and 95% (FK95), and Brownian bridge kernel 50% (BBK50) and 95% (BBK95) for giant anteaters (*Myrmecophaga tridactyla*) in 2015 in Santa Bárbara Ecological Station and its surroundings, southeast Brazil

ID	TP	Loc	Act	Inact	Dist	MCP	FK50	FK95	BBK50	BBK95
F1	10	134	39	61	88	1.43	0.79	2.61	0.13	0.92
M1	147	2,619	51	49	158	6.82	0.87	4.16	0.63	3.23
F2	46	839	48	52	131	6.30	1.18	7.27	0.32	2.57
M2	136	2,467	51	48	131	10.90	2.36	7.79	1.34	5.69
M3	93	1,608	46	54	132	6.24	1.65	6.38	0.71	4.03
F3	108	2,019	44	56	120	3.36	0.75	2.72	0.49	2.02
M4	107	2,091	44	56	173	19.50	4.03	16.55	1.40	7.90
F4	81	1,393	41	59	72	1.96	0.31	1.35	0.21	0.95
Average	—	1,646			126	7.06	1.49	6.10	0.65	3.41

**Figure 3:**
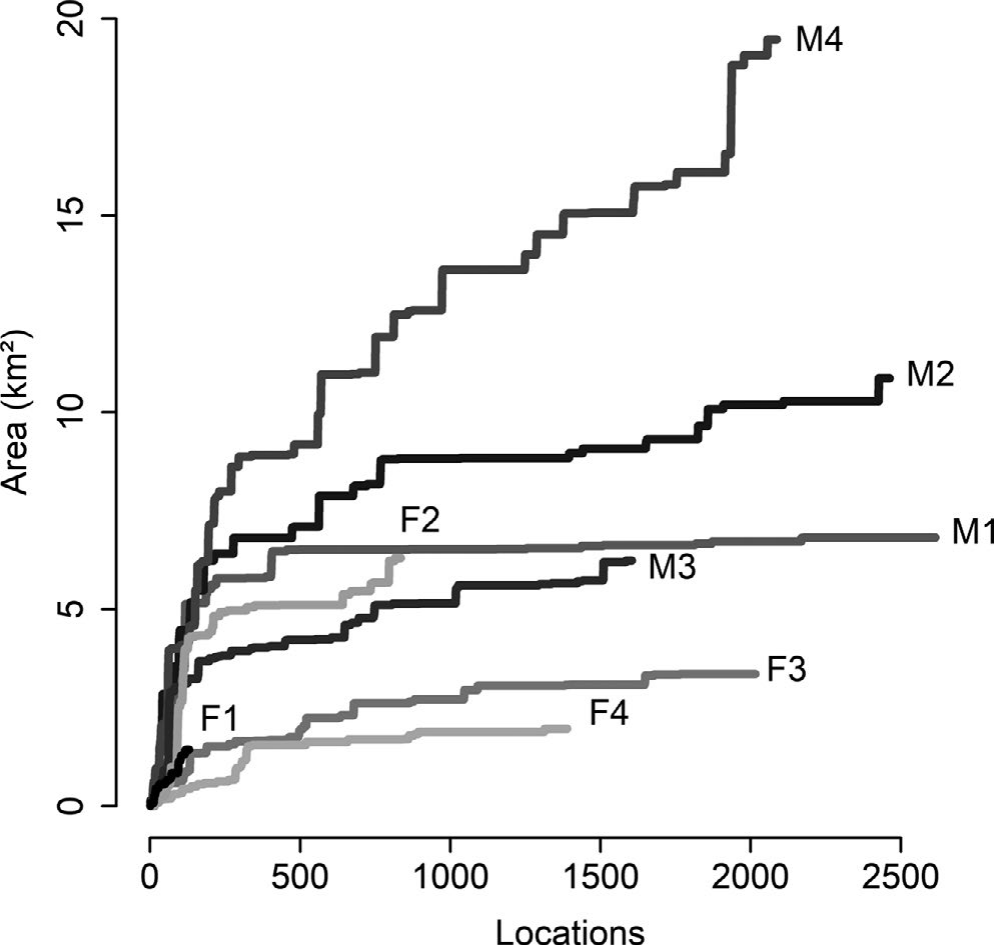
Giant anteaters (*Myrmecophaga tridactyla*) cumulative estimated home range (minimum convex polygon) tracked in 2015 at the Santa Bárbara Ecological Station and its surroundings, southeast Brazil. Written in bold is the identification of each anteater (F = female, M = male)

On average, the estimates of home range size were 3.41 km^2^ by 95% BBK, and 6.10 km^2^ by 95% FK, and 7.06 km^2^ by MCP (Table 3). The core‐areas were 0.65 km^2^ and 1.49 km^2^ using 50% KKB and FK, respectively (Table 3; Appendix S3). For anteaters with a smaller dataset (<1,000 locations), the home range stabilization curve did not reach asymptote but continued increasing. For the anteaters with a larger dataset (ranging from 1,000 and 1,500 locations), the curve tended to remain stable or slightly increasing. However, the stabilization curve of the males M2 (2,467 locations) and M4 (2091 locations) did not reach asymptote (Figure 3).

**Figure 4:**
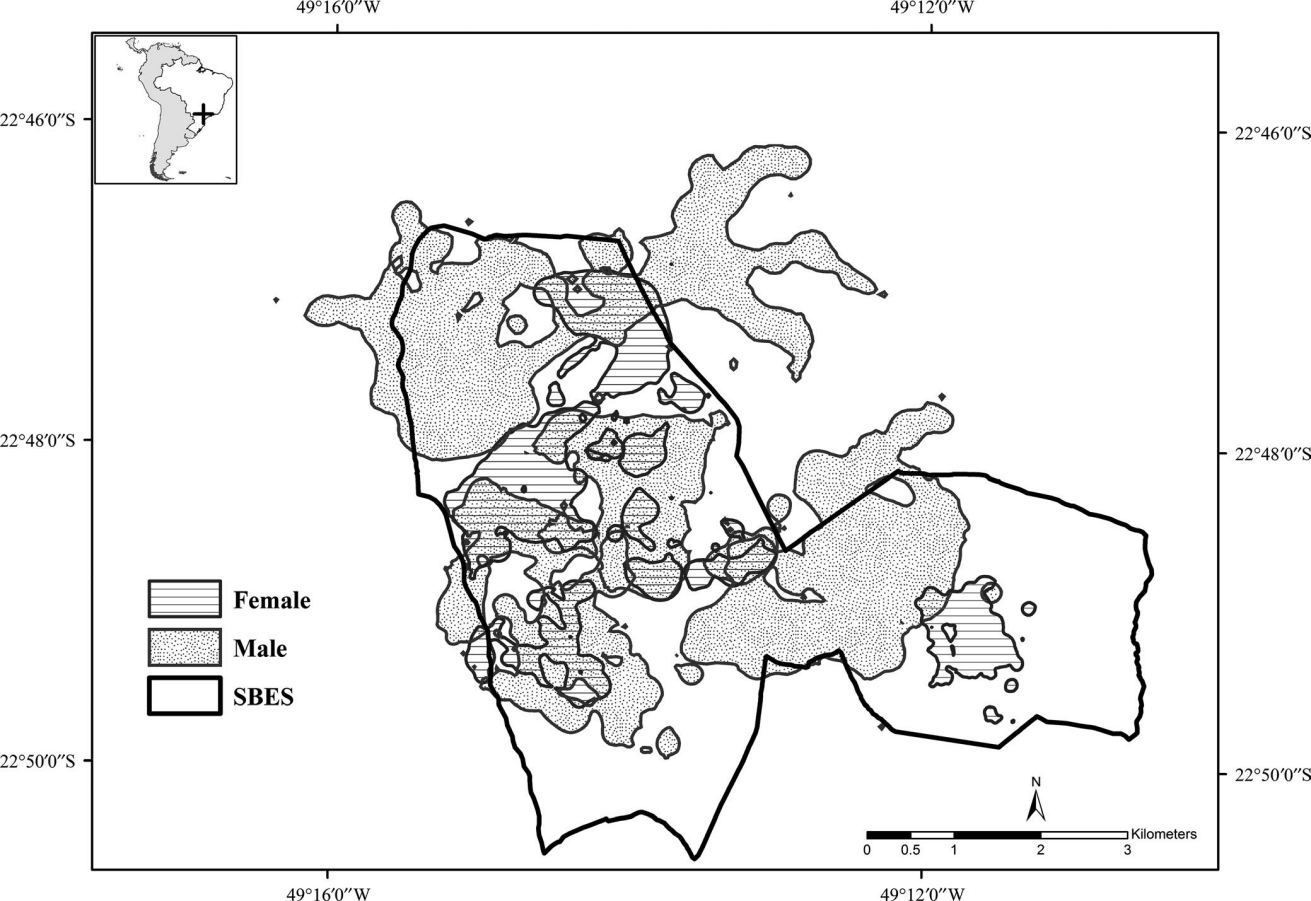
Giant anteaters (*Myrmecophaga tridactyla*) home range (Brownian bridge kernel 95%) measured in 2015 at Santa Bárbara Ecological Station and its surroundings, southeast Brazil

The BBK estimates varied according to the anteaters' sex (*t* = −3.2415, *df* = 7, *p* = .01; Table 3, Figure 4, Appendix S3). On average, the females' home range was 1.62 km^2^ and the males was 5.21 km^2^. The other estimators did not present this pattern (FK 95% *p* = .13; MCP *p* = .07). The anteaters spent more time inactive (53% of the locations) than active (*t* = −2.8093, *df* = 7, *p* = .02; Table 3), and activity time varied by sex (*t* = 33.193, *df* = 7, *p* < .01), the males being more active.

### 3.2 Home range overlap and anteaters' proximity

The probability of home range overlap (PHR) was above 40% in four dyads of anteaters (M1–F2, M3–F3, M4–F2, M4–F3) of the total of 42 possible combinations, and F2, F3, and M4 were found in more than one overlapping dyad (Table 4). The joint distribution of use (UDOI, 21 possible combinations) showed an overall low space use sharing between anteater dyads (0.01–0.18; Table 5), but the overlap between dyads of opposite sexes were larger than dyads composed by same‐sex individuals (*χ* ^2^ = 4.129, *df* = 1, *p* = .042). The proximity analysis indicated several events where two anteaters were close to each other (Table 6; Figure 6). Most of these events occurred between a male–female dyad; however, one dyad was composed of two males (M1‐M3). In some events, two individuals were around 100 m apart (Table 6).

**Table 4:** Home range overlap according to the probability of home range (PHR) index of giant anteaters (*Myrmecophaga tridactyla*) in 2015 in the Santa Bárbara Ecological Station and its surroundings, southeast Brazil

ID	M1	F2	M2	M3	F3	M4	F4
M1		0.22	0.08	0.10	0.37	0	0
F2	**0.82**		0.09	0.02	0.04	0.11	0
M2	0.05	0.04		0	0	0	0.09
M3	0.06	0.03	0		**0.54**	0.01	0
F3	0.12	0.02	0	0.17		0.04	0
M4	0.05	**0.59**	0	0.02	**0.42**		0
F4	0	0	0.09	0	0	0	

Note

Cells with PHR > 40% are highlighted in bold.

Abbreviations: F, female; M, male.

**Table 5:** Home range overlap according to utilization distribution overlap (UDOI) index of giant anteaters (*Myrmecophaga tridactyla*) in 2015 in the Santa Bárbara Ecological Station and its surroundings, southeast Brazil

ID	M1	F2	M2	M3	F3	M4	F4
M1							
F2	0.18						
M2	0	0					
M3	0.01	0	0				
F3	0.05	0	0	0.08			
M4	0	0.06	0	0	0.02		
F4	0	0	0.14	0	0	0	

Abbreviatons: F, female; M, male.

**Table 6:** Proximity of the trajectories of six dyads of giant anteater's (*Myrmecophaga tridactyla*) tracked in 2015 at the Santa Bárbara Ecological Station and its surroundings, southeast Brazil

ID	*N* ≤ 100 m	*N* ≤ 300 m	Average	<dist
M1–F2	4	44	199	76.7
M1–F3	1	13	195	100
M3–F3	15	77	196	35
M4–F2	1	2	142	106
M2–F4	0	4	255	174
M1–M3	1	7	218	80.1

Note

ID: giant anteater dyad (F, female; M, male); *N* ≤ 100 m and *N* ≤ 300 m: number of events in which the proximity was equal to or less than 100 and 300 m, respectively; average (m): average distance between the two individuals, and <dist: the shortest distance (m) between the two individuals.

### 3.3 Habitat selection

Regarding habitat availability within the study landscape, the most abundant was open habitats, followed by exotic timber plantation, savanna, forest, and anthropogenic sites (Table 1).

At 2nd Johnson order scale (landscape‐scale), the establishment of the home range (BBK95%) and core‐area (BBK50%) within the study landscape was not random (*λ* = 0.197, *df* = 4, *p* = .011; and *λ* = 0.212, *df* = 4, *p* = .014; Table 7). For the former, the giant anteaters positively selected savanna and anthropogenic sites, and for the latter, savanna was again selected, followed by open habitats. Exotic timber plantation was not selected in both, home range and core‐area. However, in the 3rd‐order analysis, habitats in the home range (*λ* = 0.378, *df* = 4, *p* = .1) and core‐area (*λ* = 0.423, *df* = 4, *p* = .147) were used randomly.

**Table 7:** Ranking matrix of habitat types selected, in the 2nd Johnson order, by giant anteaters (*Myrmecophaga tridactyla*) in 2015 in the Santa Bárbara Ecological Station and its surroundings, southeast Brazil

	For	Open	Exot	Anthr	Sava	ORDER
(A) 95% BBK home range versus landscape						
For	0					0
Open	+	0				2
Exot	+	−	0			1
Anthr	+	+	+++	0		3
Sava	+	+++	+++	+	0	4
(B) 50% BBK home range versus landscape						
For	0					2
Open	+	0				3
Exot	−	−	0			0
Anthr	−	−	+++	0		1
Sava	+	+	+++	+	0	4
(C) Active locations versus 95% BBK home range						
For	0					2
Open	+++	0				3
Exot	−	−	0			0
Anthr	−	−	+	0		1
Sava	+++	+	+	+	0	4

Note

The last column shows the habitat selection ordination. (A) Proportional habitat use within 95% Brownian bridge kernel (BBK) home ranges, with proportion of total available habitat types within study site; (B) proportional habitat use within 50% BBK home ranges, with proportion of total available habitat types within study site; (C) proportions of giant anteaters' active locations in each habitat type within 95% BBK home ranges. Triple symbols (+++) represents significant deviation from random at *p* < .05. A single symbol (+) indicates that the habitat was positively selected.

For, forest; Open, open habitats; Exot, exotic timber plantation; Anthr, anthropogenic sites; Sava, savanna.

**Figure 5:**
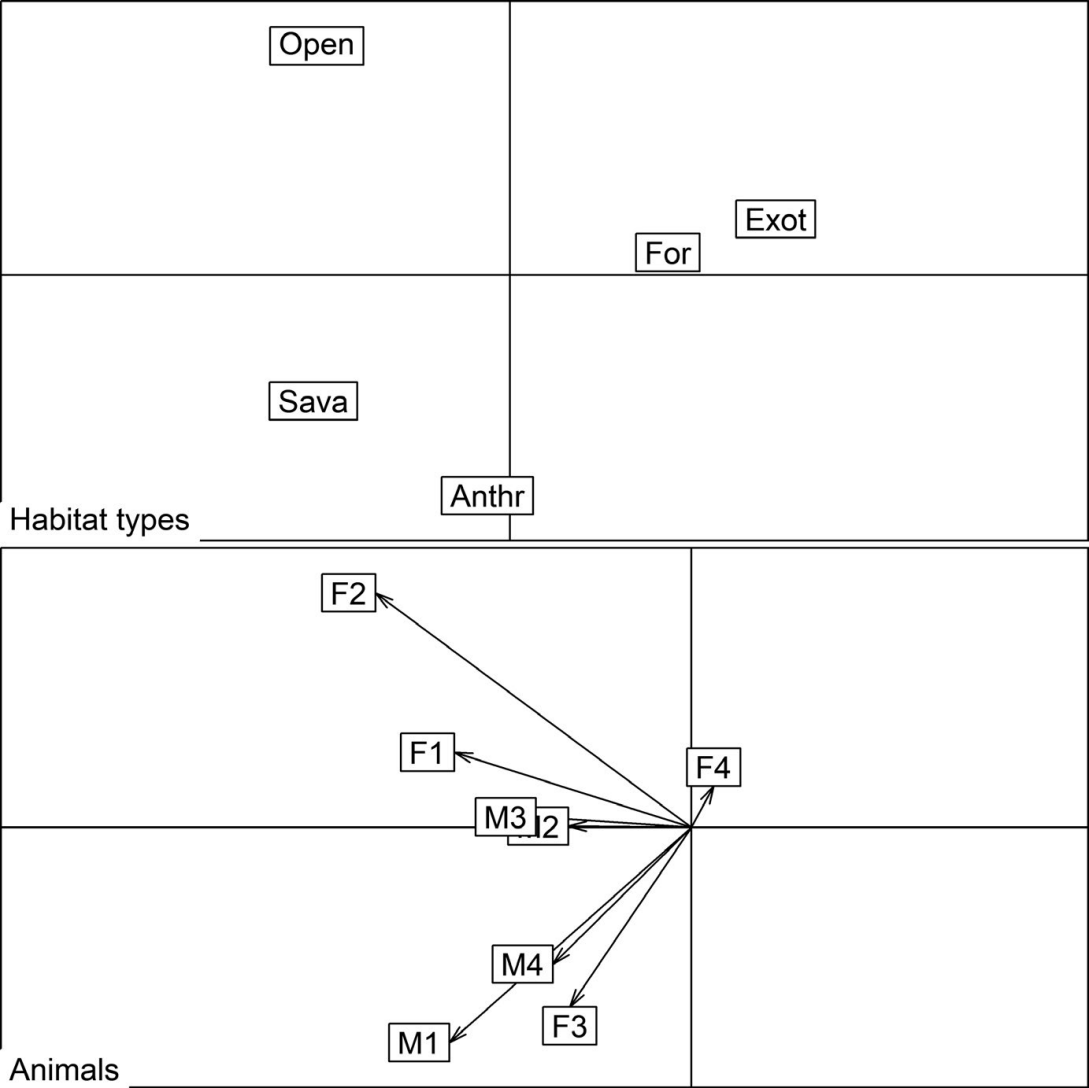
Results of *eigen*‐analysis in the 3rd order of Johnson for active giant anteaters (*Myrmecophaga tridactyla*) at Santa Bárbara Ecological Station and its surroundings, southeast Brazil. For = forest; Open = open habitats; Exot = exotic timber plantation; Anthr = anthropogenic sites; and Sava = savanna

While active, anteaters did not use habitat types randomly under the 95% BBK model (3rd Johnson order *λ* = 0.174, *df* = 4, *p* = .007; Figure 5, Table 7), and savanna and open habitats were positively selected. However, habitat use was random within the core‐areas when the anteaters were active (*λ* = 0.702, *df* = 4, *p* = .588), and in both the home range (*λ* = 0.498, *df* = 4, *p* = .2) and the core‐area (*λ* = 0.429, *df* = 4, *p* = .1) when the anteaters were inactive.

**Figure 6:**
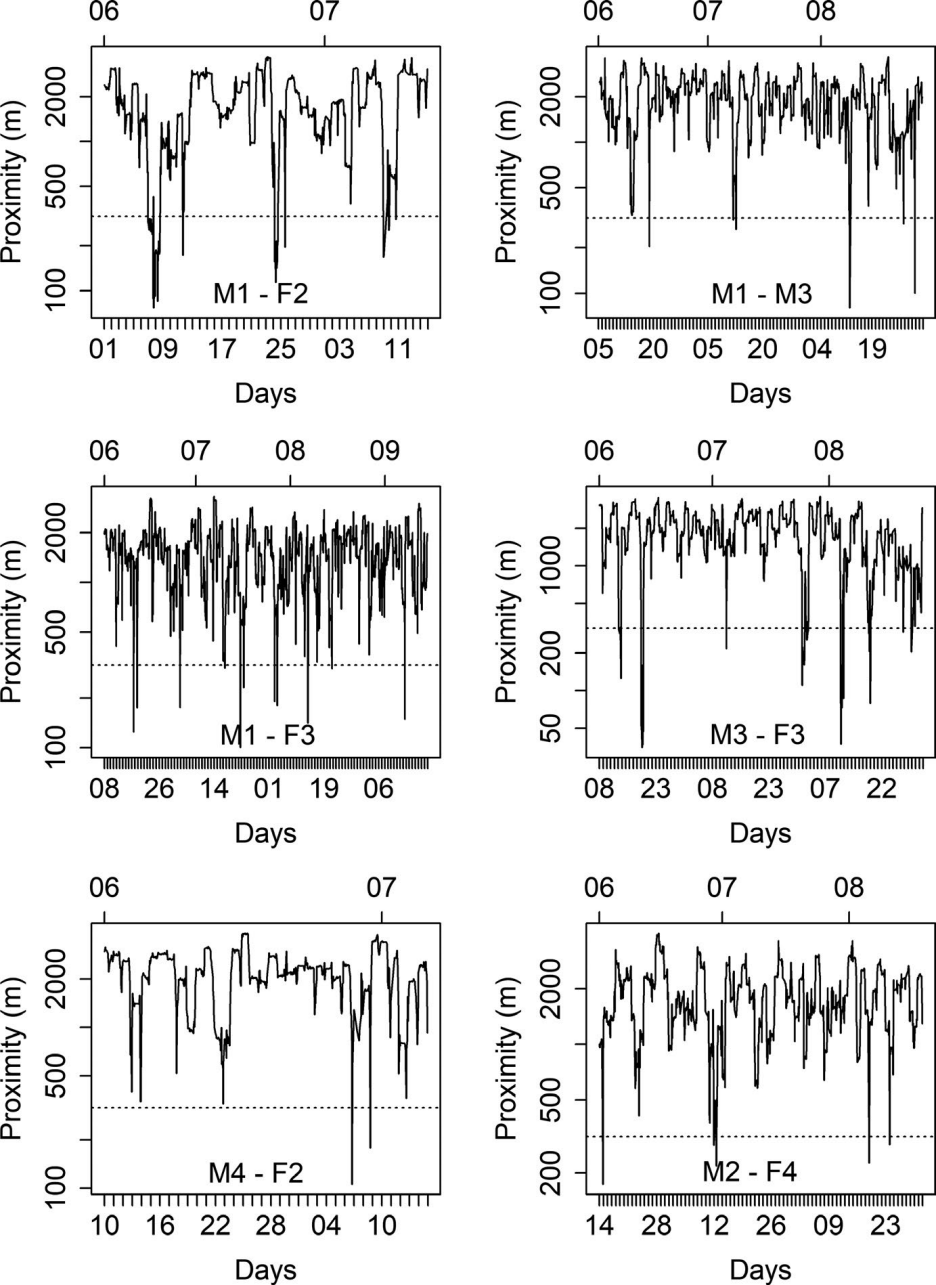
Proximity of the trajectories of six dyads of giant anteaters (*Myrmecophaga tridactyla*) in 2015 in the Santa Bárbara Ecological Station and its surroundings, southeast Brazil. The *x*‐axis represents consecutive days, the parallel axis above shows the month (06 June, 07 July, and 08 August), and the y‐axis shows the logarithmic distance (meters) in which the event occurred. F = female and M = male. The dotted line is the proximity threshold of 300 m

## 4 DISCUSSION

### 4.1 Tracking and home range

This is the first published study of giant anteaters monitored by GPS transmitters in an extensively human‐modified landscape. The studied anteaters were resident within SBES, but the home ranges of two individuals extended outside it (M2 and M4). Those two males also had the largest home ranges, with an increasing trend (Table 3; Figure 2). This suggests the presence of some suitable areas outside the SBES to be exploited, despite the risks associated with the anthropogenic actions in such human‐modified area. A similar pattern was already found on male pumas (*Puma concolor*) in patches of Cerrado remnant in the São Paulo State (Miotto, Cervini, Begotti, & Galetti, 2012). However, the only female that had locations outside the protected area disappeared, and the circumstances pointed to a hunting event (Bertassoni et al., 2017). Although the use of the landscape outside protected areas may supply individual requirements for the maintenance of viable populations of wild animals (Santini et al., 2013), it may be particularly risky for individuals that use sites in a human‐modified landscape influenced by anthropogenic actions (Bertassoni et al., 2017; Clobert, Le Galliard, Cote, Meylan, & Massot, 2009; Miotto et al., 2012).

Our results showed that the anteaters' home ranges varied widely in size, and this seems to be caused by internal and external factors, even for multiple individuals tracked concurrently in the same site. The degree of anthropogenic influence on a landscape has to be considered among these factors (Rodrigues, Medri, Miranda, Camilo‐Alves, & Mourão, 2008). The study landscape is human‐modified and influenced by human actions, particularly near the SBES boundaries. The anteaters whose home ranges include these boundaries are likely to be more susceptible to anthropogenic impacts due to the proximity of land‐use change, roads, waste deposits, etc. Nevertheless, the effect of anthropogenic influence on the variation in home range size is still poorly understood.

Males were more active than females, making them easier to observe and therefore capture. This higher activity may influence the establishment of their home range and leading them to have larger home ranges than the females do. This could also explain the slightly male‐biased sex ratio found in this study, although it has been documented also in previous studies (Camilo‐Alves & Mourão, 2006; Medri & Mourão, 2005; Miranda, 2004; Rodrigues et al., 2003; Shaw et al., 1987). However, the species' natural sex ratio at birth could not be dismissed as a factor, though the lack of reproductive surveys in the wild obscures further insight on the matter (Miranda et al., 2015; Superina, Miranda, & Abba, 2010).

### 4.2 Home range overlap and anteaters' proximity

Home range overlaps among giant anteaters based just in spatial overlap were documented elsewhere and were in general high, ranging from 55% to 100% (Macedo et al., 2010; Medri & Mourão, 2005; Miranda, 2004). Here, we used two novel approaches to assess the overlap (UDOI and PHR, Fieberg & Kochanny, 2005), though these overlapping home ranges estimates were not directly comparable to the formers. Nonetheless, our results suggested a general low degree of spatial sharing (Tables 4 and 5), although dyads composed by opposite sexes overlap more than same‐sex dyads. Our proximity analysis suggests that some dyads of individuals were frequently predisposed toward coexistence. Approximations occurred seven times for a dyad of males and repeatedly for opposite‐sex dyad. For example, one of the male–female dyads (M3‐F3) was repeatedly found at very short distances from each other between June and August (Table 6; Figure 5). This indicates possible reproductive behavior, considering that the estrus period lasts seven weeks (from 55 to 74 days) and that females can have multiple interactions with males during that period (Gaudin, Hicks, & Di Blanco, 2018; Knott et al., 2013). However, the species' reproductive seasonality is still largely unknown (Gaudin et al., 2018; Redford, 1994). There are also evidences of aggressive behavior between dyads of individuals of unknown sex (Kreutz, Fischer, & Linsenmair, 2009; Rocha & Mourão, 2006; Shaw et al., 1987) and between a male–female dyad (Miranda & Bertassoni, 2014). Although these proximity events indicate a higher level of coexistence than previously reported for this supposed solitary species, the results of these analyses highlight that low home range overlaps cannot be strongly interpreted as an indication of lack of individuals' interaction. For example, despite the overall low home ranges overlap, six dyads had been relatively close proximity during our study (five of these composed of opposite sexes and one of males).

### 4.3 Habitat selection

Savanna was positively selected for the establishment of the home range and core‐area, in the landscape as well as within the home range scales, when active. This habitat has a heterogeneous shrub‐tree structure and discontinuous grass stratum; therefore, it may provide shelter during daily temperature extremes, which is an important environmental factor for giant anteaters (Camilo‐Alves & Mourão, 2006; Di Blanco, Jimenez‐Perez, & Di Bitetti, 2015; Mourão & Medri, 2007). The SBES is a mosaic of different vegetation physiognomies (Melo & Durigan, 2011), in which open habitats are directly exposed to climatic conditions, and forests are covered habitats. Our results show that habitat selection favored an intermediate habitat in terms of climatic exposure. At the landscape level (2nd Johnson order), the second most selected habitat was anthropogenic areas. The SBES has numerous dirt roads (firebreaks) that facilitate access, and the surrounding areas also have many unpaved accesses. Our data showed locations in those areas (Appendix S2), and unpaved roads and accesses probably increase the anteaters' ability to travel there. In the south of Brazil, in an exotic timber plantation, two giant anteaters positively selected roads (Braga, 2010). Other studies have also reported the use of dirt roads and highway shoulders (Freitas, Justino, & Setz, 2014; Macedo et al., 2010; Vynne et al., 2011), and giant anteater occupancy was recently connected to dirt roads in Cerrado areas in São Paulo State (Versiani, 2016). In contrast, another study reported that the giant anteater was rarely observed in anthropogenic areas of a Cerrado remnant in the same state (Lyra‐Jorge & Pivello, 2005); however, this particular Cerrado remnant (90 km^2^) is the largest in São Paulo, and its resources may enable the use of more natural habitats.

The exotic timber plantation was avoided in both orders of habitat selection. The locations recorded in this habitat were probably related to the individuals' passage (use in movement). Mammals using exotic timber plantations for passage were documented in other Cerrado areas of São Paulo (Lyra‐Jorge & Pivello, 2005). However, despite the use of habitats in heavily modified landscapes dominated by exotic species, habitat selection is still associated with the natural habitats (Braga, 2010; Kreutz, Fischer, & Linsenmair, 2012; Timo, Lyra‐Jorge, Gheler‐Costa, & Verdade, 2015; Vynne et al., 2011).

The sum of all locations in our dataset showed that the anteaters spent more of their time being inactive rather than active. This pattern, already documented elsewhere, may be a behavioral response to the species' low metabolic rate, which limits it energetic expenditure (Camilo‐Alves & Mourão, 2006; Macedo et al., 2010; McNab, 1984). Therefore, an ecological response is also expected, and it can appear in the habitat selection. We found that at the home range, core‐area and activity levels, the anteaters primarily selected the savanna, a type of habitat able to meet their thermoregulatory needs. A pattern of habitat selection associated with activity and thermoregulation was also found in the Pantanal wetland, where the anteaters used predominantly forest habitats for rest and open habitats for activity (Camilo‐Alves & Mourão, 2006; Mourão & Medri, 2007). In our study site, however, we did not find a habitat selection pattern for inactive anteaters, probably because the habitat used for rest was not as important as the habitat used when active. The ranking of habitats selected during activity and within the core‐area showed a pattern of preference for more open habitats. Rojano‐Bolaño, Giraldo, Miranda‐Cortés, and Avilán (2015) in Colombia also found a selection for open natural savanna. Possibly, savanna and open habitats have a higher productivity of anteater prey (ants and termites), an essential feature because the time spent in activity is mainly devoted to finding and consuming prey (Bertassoni & Costa, 2010).

Anthropogenic sites were frequently selected at the landscape level (2nd Johnson order), reinforcing an adaptation trend related to the giant anteaters' habitat use, as documented in other areas (Braga, 2010; Kreutz et al., 2012; Miranda, 2004; Vynne et al., 2011). Nevertheless, our results also reveal the importance of natural habitats at all analyzed levels. This established dichotomy shows that habitat plasticity has limitations, and it highlights the strong dependence on natural habitats in the space use of giant anteaters in habitat patches surrounded by human‐modified landscape. The anteaters' habitat needs may affect their ability to establish their home ranges in such patches and compel some individuals to intensify their exploratory behavior. The risk of removal is high for individuals exploring the environment in a human‐modified landscape, though, and may lead to local population decline and loss of genetic variability (Diniz & Brito, 2015; Ribeiro, 2016). Thus, it is important to maintain and protect native patches near protected areas and to effectively apply guidelines concerning protected area zoning and its use.

### 4.4 Final considerations

Our results indicate that the BBK responds to biologically important requirements when determining home range. The trajectory of such specialized insectivorous is likely shaped by the prey distribution in the landscape (Montgomery & Lubin, 1977). Thus, a trajectory‐based home range will probably provide more information about the distribution and abundance of preys than the probabilities home ranges methods like FK. To the best of our knowledge, this study is the first to address trajectory‐based home range estimation for giant anteaters. We also provide information on habitat selection at three scales in two orders of selection, which highlights important patterns of space use for the species in a human‐modified landscape.

Currently, the entire Cerrado biome—a world biodiversity hotspot—is extremely fragmented, persisting only in remnants within governmentally and privately protected areas and in sites where agriculture and other economic activities are not sustainable (e.g., due to relief and slope; Beuchle et al., 2015; Durigan et al., 2007). This is the context of the landscape where the SBES is situated, hence its importance for biodiversity conservation, as this small protected island of Cerrado vegetation is able to maintain some wild species, such as the giant anteater. However, because the carrying capacity of areas like the SBES is limited (Diniz & Brito, 2015), environmentally friendly management practices are essential in the neighboring areas, especially those including agriculture, timber plantation, and pasture to increase the wildlife carrying capacity of anthropogenic landscapes (Verdade et al., 2014). In addition, we recommend wildlife management at the population level, such as sustainable use of the landscape and wildlife monitoring.

## CONFLICT OF INTEREST

None declared.

## AUTHOR CONTRIBUTIONS

AB and RCB conceived and designed the study. AB collected all the data and created the maps and figures. AB and GM performed the analysis. AB wrote the manuscripts, and GM and RCB revised it critically. All the authors gave final approval for publication.

## Data Availability

Partial data used in this study are available at: Github repository—https://github.com/LEEClab/Neotropical_Series.
