# Supplementary material for: Space use by giant anteaters (Myrmecophaga tridactyla) in a protected area within human‐modified landscape
Source: Ecol Evol. 2020 Jul 13;10(15):7981–94. doi: 10.1002/ece3.5911 (PMC7417248; doi:10.1002/ece3.5911)

**Supporting Information** **1.** Giant anteaters (*Myrmecophaga tridactyla*) captured in 2015 at Santa Bárbara Ecological Station and its surroundings, Southeast Brazil. Each frame is represents one of the giant anteaters studied.


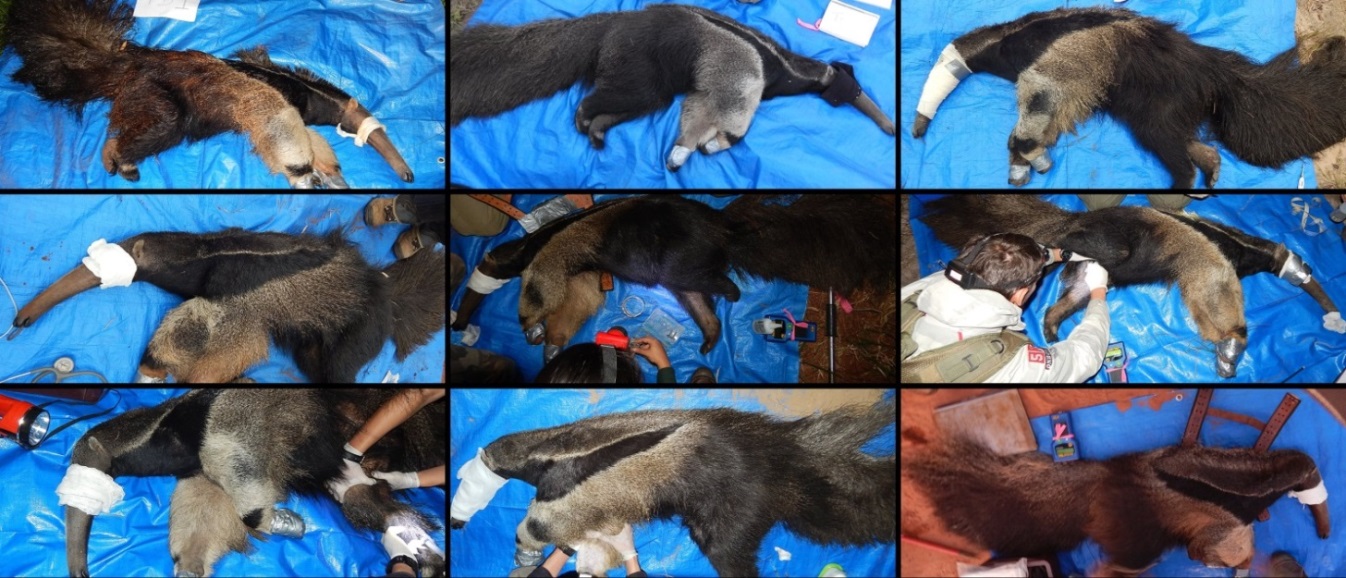

Supplement: Supplementary file 1 [file ECE3-10-7981-s001.docx]
