# Supplementary material for: Space use by giant anteaters (Myrmecophaga tridactyla) in a protected area within human‐modified landscape
Source: Ecol Evol. 2020 Jul 13;10(15):7981–94. doi: 10.1002/ece3.5911 (PMC7417248; doi:10.1002/ece3.5911)

**Supporting Information 2**. Santa Bárbara Ecological Station and its surroundings in Southeast Brazil. The map shows the giant anteaters’ (*Myrmecophaga tridactyla*) locations. (F = female, M = male).


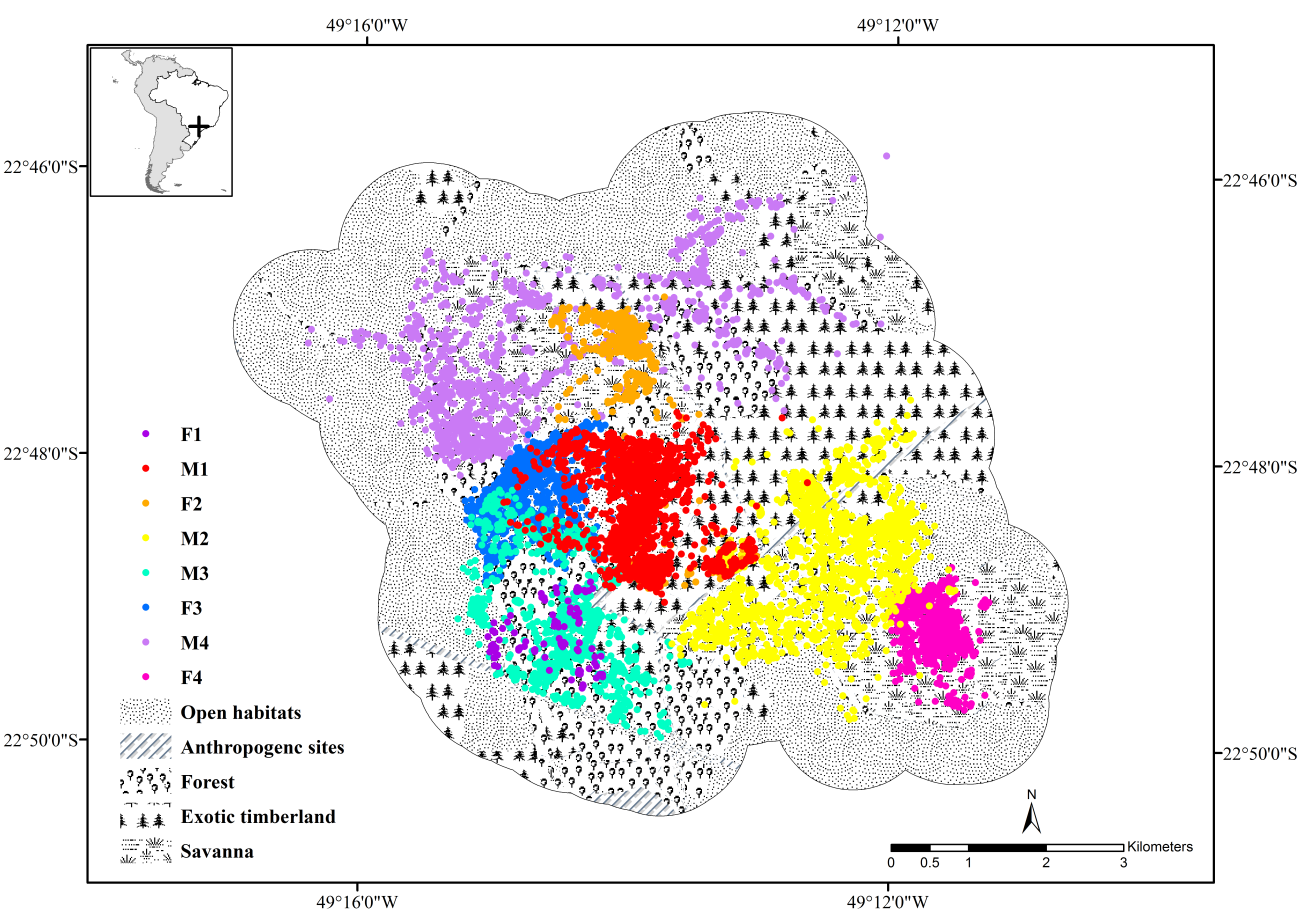

Supplement: Supplementary file 2 [file ECE3-10-7981-s002.docx]
