# Supplementary material for: Space use by giant anteaters (Myrmecophaga tridactyla) in a protected area within human‐modified landscape
Source: Ecol Evol. 2020 Jul 13;10(15):7981–94. doi: 10.1002/ece3.5911 (PMC7417248; doi:10.1002/ece3.5911)

**Supporting Information 3.** The relationship between the giant anteaters’ (*Myrmecophaga tridactyla*) locations and home range, according to sex (F = female, M = male), in the Santa Bárbara Ecological Station and its surroundings, Southeast Brazil.


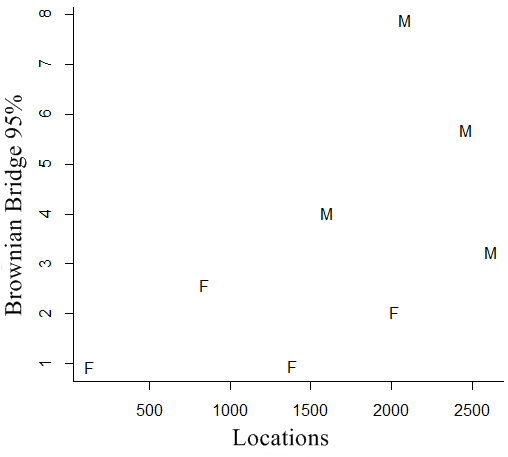

Supplement: Supplementary file 3 [file ECE3-10-7981-s003.docx]
